# Immunomodulation in healing: neoadjuvant immunochemotherapy reduces major wound complications and accelerates recovery in oral cancer surgery

**DOI:** 10.3389/fimmu.2026.1768004

**Published:** 2026-02-27

**Authors:** Hongli Chen, Yang Shi, Qigen Fang, Lijie Hu

**Affiliations:** 1 Department of Stomatology, Henan People’s Hospital, Zhengzhou University People’s Hospital, Zhengzhou, Henan, China; 2 Department of Head Neck, The Affiliated Hospital of Zhengzhou University & Henan Cancer Hospital, Zhengzhou, China

**Keywords:** neoadjuvant immunotherapy, oral squamous cell carcinoma, pathological response, surgical complications, wound healing

## Abstract

**Background:**

Major wound complications after oral squamous cell carcinoma surgery remain a significant cause of morbidity, delaying adjuvant therapy and impairing recovery. Neoadjuvant immunochemotherapy may improve pathological responses, but its impact on postoperative wound healing is poorly understood. This study evaluates whether neoadjuvant immunochemotherapy is associated with fewer major wound complications and faster healing compared to neoadjuvant chemotherapy or upfront surgery.

**Methods:**

In this retrospective cohort study, 692 patients with locally advanced oral squamous cell carcinoma undergoing curative-intent surgery were divided into three cohorts: neoadjuvant immunochemotherapy (n=235), neoadjuvant chemotherapy (n=300), and upfront surgery (n=157). The primary endpoint was incidence of major wound complications (Clavien-Dindo ≥III) within 90 days. Secondary endpoints included time to complete wound sealing, length of postoperative stay, readmissions, reoperations, and time to adjuvant radiotherapy.

**Results:**

Major wound complication rates were significantly lower in the neoadjuvant immunochemotherapy cohort (9.2%) compared to neoadjuvant chemotherapy (17.8%) and upfront surgery (21.5%) (p<0.001). Multivariable analysis confirmed neoadjuvant immunochemotherapy was independently associated with reduced complication risk versus upfront surgery (adjusted odds ratio 0.40, p=0.001) and neoadjuvant chemotherapy (adjusted odds ratio 0.48, p=0.007). Time to complete wound sealing was significantly shorter in the neoadjuvant immunochemotherapy cohort (adjusted hazard ratio 1.85 vs. upfront surgery, p<0.001). Neoadjuvant immunochemotherapy patients also had shorter hospital stays, fewer readmissions, and earlier initiation of adjuvant radiotherapy. Within the neoadjuvant immunochemotherapy cohort, poor pathological response, shorter treatment-surgery interval (<28 days), heavy smoking, free flap reconstruction, and high blood loss were independent risk factors for complications.

**Conclusions:**

Neoadjuvant immunochemotherapy is associated with a significant reduction in major wound complications and accelerated wound healing in patients with locally advanced oral squamous cell carcinoma surgery, supporting its integration into perioperative care pathways to improve surgical recovery.

## Introduction

Oral squamous cell carcinoma (OSCC) remains a significant global health burden, with over 350,000 new cases diagnosed annually worldwide (1). For patients with locally advanced, yet resectable disease, the standard of care has traditionally involved multimodal therapy, primarily upfront surgery followed by adjuvant radiotherapy or chemoradiotherapy (2). Despite advances in surgical techniques, postoperative morbidity, particularly related to wound healing in the complex head and neck region, continues to be a major challenge (3). Major wound complications occur in 15-30% of cases, leading to prolonged hospital stays, delays in adjuvant therapy, increased healthcare costs, and diminished quality of life. These complications are influenced by a confluence of patient-related factors such as malnutrition, comorbidities, smoking, tumor-related factors including size and location, and treatment-related factors, most notably the extent of surgical resection and reconstruction (4).

In an effort to improve oncological outcomes, neoadjuvant chemotherapy (NCT) has been extensively investigated in locally advanced OSCC. While NCT can achieve tumor downstaging, its impact on survival has been inconsistent, and concerns have been raised regarding its potential to increase perioperative toxicity and impair wound healing due to systemic cytotoxic effects on cellular proliferation and immune function (5). The recent integration of immunotherapy into the neoadjuvant paradigm represents a transformative shift. Neoadjuvant immunochemotherapy (NICT) leverages the ability of immune checkpoint inhibitors to activate tumor-specific T-cells, potentially enhancing pathological response rates and generating a durable systemic immune response (6). Emerging data from several solid tumors suggest that neoadjuvant immunotherapy may not only improve tumor regression but could also favorably modulate the postoperative wound microenvironment, possibly leading to enhanced healing (7–9).

The immunomodulatory effects of NICT present a compelling, yet unexplored, hypothesis in OSCC surgery. Unlike traditional chemotherapy, which is broadly immunosuppressive, immunotherapy may prime the immune system for a more coordinated repair response. A robust pathological response, characterized by significant tumor cell death and immune infiltration, reduces the local tumor burden, which may alleviate lymphatic obstruction and improve tissue perfusion. Furthermore, preclinical evidence suggests that immune checkpoint blockade can promote a shift toward a pro-reparative M2 macrophage phenotype, theoretically reducing the inflammatory burden and creating a more favorable milieu for healing (10). However, these potential benefits must be rigorously balanced against the risk of immune-related adverse events, which could theoretically impede healing. To date, no comprehensive study has specifically evaluated the impact of NICT on surgical wound healing outcomes in patients with OSCC.

Therefore, we aimed to evaluate the incidence and predictors of major wound complications in patients with locally advanced oral squamous cell carcinoma undergoing curative-intent surgery, comparing three distinct preoperative pathways: neoadjuvant immunochemotherapy, neoadjuvant chemotherapy, and upfront surgery (US). We hypothesized that neoadjuvant immunochemotherapy would be associated with a lower rate of major wound complications and accelerated time to complete wound healing compared to both neoadjuvant chemotherapy and upfront surgery. By identifying modifiable risk factors and optimal treatment intervals, this study seeks to provide clinically actionable insights to optimize perioperative management and improve recovery for patients receiving modern neoadjuvant immunochemotherapy.

## Methods

### Study design and setting

This is a single-center, retrospective cohort study conducted at Henan People’s Hospital to evaluate the impact of NICT on postoperative wound healing in patients with locally advanced OSCC. The study protocol was approved by the Henan People’s Hospital Institutional Review Board, an informed written consent was obtained from all patients.

### Patient selection and cohort definitions

Patient selection began with identification through the institutional head and neck cancer registry and pharmacy databases between January 2015 and December 2025. The inclusion criteria were: (1) histologically confirmed primary OSCC; (2) clinical stage III-IVB (AJCC 8th edition) deemed resectable by multidisciplinary tumor board; (3) underwent curative-intent surgery with neck dissection; and (4) age ≥18 years. Three cohorts were then defined based on preoperative treatment: the NICT Cohort, comprising patients who received NICT followed by surgery; the NCT Cohort, a historical control group who received neoadjuvant chemotherapy (platinum-based doublet or triplet) without immunotherapy, followed by surgery; and the US Cohort, a baseline control group who underwent primary surgery without any neoadjuvant systemic therapy. Key exclusion criteria applied to all groups were: (1) distant metastasis at diagnosis; (2) prior radiotherapy to the head and neck region; (3) synchronous malignancies; (4) incomplete medical records or loss to follow-up within 90 days post-surgery; and (5) salvage surgery for recurrent disease.

### Data collection and variables

Data were collected from electronic medical records using a standardized, piloted data collection form, encompassing a comprehensive set of variables. These included demographics and baseline characteristics (age, sex, body mass index, smoking pack-years, alcohol use, and comorbidities quantified via the Charlson Comorbidity Index), tumor characteristics (primary subsite, clinical and pathological T/N stage per AJCC 8th edition, and differentiation grade), and detailed neoadjuvant treatment information (regimen, number of cycles, immune-related adverse events, corticosteroid use, and the treatment-to-surgery interval). Surgical details were also recorded, including the date of surgery, type of resection and neck dissection, reconstructive method, flap ischemia time, total operative time, estimated blood loss, and operating surgeon. Pathological response was centrally assessed by dedicated head and neck pathologists using the Mandard Tumor Regression Grade and percentage of viable tumor cells, with major Pathological Response defined as ≤10% viable tumor. Postoperative wound healing outcomes, constituting the study’s primary and secondary endpoints, were ascertained through a systematic 90-day review of daily nursing notes, physician progress notes, drain output logs, and procedure notes.

### Study endpoints

The study defined its primary endpoint as the incidence of major wound healing complications within 90 days post-surgery, where a major complication was defined by the requirement for procedural or surgical intervention, corresponding to a Clavien-Dindo Classification of Grade ≥ III. This specifically encompassed orocutaneous or pharyngocutaneous fistula requiring interventional drainage or surgery, wound dehiscence necessitating return to the operating room, partial or total flap necrosis requiring surgical debridement or revision, deep/organ space surgical site infection requiring radiological or operative drainage, and hematoma or bleeding requiring evacuation. Secondary endpoints were established for further analysis, including the time to complete wound sealing, defined as the number of days from surgery until the first day on which both of the following criteria were met: (1) removal of the last surgical drain, and (2) the wound was documented as “closed,” “sealed,” “dry,” or “without drainage” on two consecutive assessments. Patients who developed a fistula or major dehiscence before meeting these criteria were censored at the time of the complication. Additional secondary endpoints comprised postoperative length of hospital stay, 30-day readmission rate, unplanned reoperation rate, and time to initiation of adjuvant radiotherapy.

### Statistic analysis

Statistical analysis involved presenting continuous variables as mean ± standard deviation or median with interquartile range (IQR), compared using Student’s t-test, Mann-Whitney U test, or ANOVA, while categorical variables were expressed as frequencies and percentages and compared using Chi-square or Fisher’s exact tests. For the primary endpoint, the incidence of major wound complications was compared using conditional logistic regression, supplemented by a multivariable logistic regression on the full cohort to identify independent risk factors, including treatment interval and pathological response. Time-to-event analysis for wound sealing involved plotting Kaplan-Meier curves, censoring patients who developed a major complication on that day or who lacked the event by day 90, with comparisons made via the log-rank test and hazard ratios derived from an adjusted Cox proportional-hazards model. Variables for the multivariable logistic and Cox regression models were selected based on clinical relevance and a univariate significance threshold of p < 0.10. All selected variables were entered simultaneously using the enter method. The proportional hazards assumption for the Cox regression model was assessed using Schoenfeld residual tests, and no significant violations were detected (global test p = 0.412). Planned subgroup and sensitivity analyses included evaluating complication rates by neoadjuvant-surgery intervals, assessing the correlation between pathological response and wound complications, repeating the primary analysis with a strict clinical healing definition, and identifying risk factors within the NICT cohort. A two-sided p-value below 0.05 was deemed statistically significant, with all analyses performed using R version 4.3.0 and SPSS version 26.0.

## Results

### Baseline data

The NICT cohort comprised 235 patients, with a mean age of 58.4 ± 9.2 years; the NCT cohort included 300 patients (mean age 61.1 ± 8.8 years); and the US cohort had 157 patients (mean age 59.7 ± 10.1 years). Sex distribution was comparable across groups (p=0.283). The median smoking pack-years differed significantly (p<0.001), being highest in the NCT cohort (30 pack-years) and lowest in the US cohort (20 pack-years). The Charlson Comorbidity Index distribution showed a statistically significant difference among cohorts (p=0.045). Significant differences were observed in tumor stage distribution. The NICT and NCT cohorts had more advanced clinical T and N stages compared to the US cohort (p<0.001 for both). Consequently, the clinical stage grouping also differed significantly (p<0.001), with a higher proportion of stage IVA–IVB disease in the neoadjuvant groups. Tumor differentiation grade was not significantly different across cohorts (p=0.118). In the neoadjuvant groups, the median number of cycles was 3 for both. However, the treatment-to-surgery interval was longer in the NICT cohort (median 28 days) compared to the NCT cohort (median 21 days). Surgical complexity varied among cohorts, with significant differences in reconstructive method (p=0.012), total operative time (p=0.001), and estimated blood loss (p<0.001). The NICT cohort had a higher proportion of free flap reconstructions and longer operative times. Among patients receiving neoadjuvant therapy, pathological outcomes differed markedly. The NICT cohort demonstrated superior response rates, with 29.8% achieving a pCR and 57.4% achieving a mPR, compared to only 4.0% pCR and 15.0% mPR in the NCT cohort (p<0.001). Mandard Tumor Regression Grade and the percentage of viable tumor cells also showed significantly better responses in the NICT group (p<0.001) (**Table 1**).

**Table 1:** Baseline characteristics of the study cohorts.

Characteristic	NICT Cohort (n=235)	NCT Cohort (n=300)	US Cohort (n=157)	p-value
Demographics and Clinical characteristics				
Age (years), mean ± SD	58.4 ± 9.2	61.1 ± 8.8	59.7 ± 10.1	0.071
Sex, n (%)				0.283
Male	185 (78.7)	225 (75.0)	125 (79.6)	
Female	50 (21.3)	75 (25.0)	32 (20.4)	
BMI (kg/m²), mean ± SD	22.3 ± 3.1	21.8 ± 3.4	23.0 ± 3.5	0.065
Smoking (pack-years), median (IQR)	25 (10–40)	30 (15–45)	20 (5–35)	<0.001
Alcohol use, n (%)	155 (66.0)	210 (70.0)	95 (60.5)	0.102
Charlson Comorbidity Index, n (%)				0.045
0–1	145 (61.7)	165 (55.0)	105 (66.9)	
2–3	75 (31.9)	105 (35.0)	45 (28.7)	
4	10 (4.3)	21 (7.0)	5 (3.2)	
≥5	5 (2.1)	9 (3.0)	2 (1.3)	
Tumor characteristics				
Primary Tumor Site, n (%)				0.210
Oral tongue	90 (38.3)	105 (35.0)	65 (41.4)	
Buccal mucosa	40 (17.0)	60 (20.0)	25 (15.9)	
Floor of mouth	45 (19.1)	50 (16.7)	30 (19.1)	
Alveolar ridge	35 (14.9)	50 (16.7)	22 (14.0)	
Retromolar trigone	25 (10.6)	35 (11.7)	15 (9.6)	
Clinical T Stage (AJCC 8th), n (%)				<0.001
T1–T2	40 (17.0)	75 (25.0)	80 (51.0)	
T3	90 (38.3)	120 (40.0)	50 (31.8)	
T4	105 (44.7)	105 (35.0)	27 (17.2)	
Clinical N Stage (AJCC 8th), n (%)				<0.001
N0	50 (21.3)	75 (25.0)	70 (44.6)	
N1	65 (27.7)	90 (30.0)	40 (25.5)	
N2	110 (46.8)	120 (40.0)	45 (28.7)	
N3	10 (4.3)	15 (5.0)	2 (1.3)	
Clinical Stage Group, n (%)				<0.001
III	100 (42.6)	135 (45.0)	95 (60.5)	
IVA	120 (51.1)	135 (45.0)	55 (35.0)	
IVB	15 (6.4)	30 (10.0)	7 (4.5)	
Tumor Differentiation, n (%)				0.118
Well	50 (21.3)	75 (25.0)	40 (25.5)	
Moderate	125 (53.2)	150 (50.0)	85 (54.1)	
Poor	60 (25.5)	75 (25.0)	32 (20.4)	
Neoadjuvant treatment details				
Number of Cycles, median (IQR)	3 (2–4)	3 (2–3)	–	–
Treatment-to-Surgery Interval (days), median (IQR)	28 (21–35)	21 (18–28)	–	–
Surgical details				
Neck Dissection, n (%)				0.425
Selective	140 (59.6)	180 (60.0)	100 (63.7)	
Modified radical	95 (40.4)	120 (40.0)	57 (36.3)	
Reconstructive Method, n (%)				0.012
Primary closure	50 (21.3)	75 (25.0)	55 (35.0)	
Local/regional flap	100 (42.6)	135 (45.0)	65 (41.4)	
Free flap	85 (36.2)	90 (30.0)	37 (23.6)	
Flap Ischemia Time (min), mean ± SD	82.5 ± 15.3	85.0 ± 18.2	80.1 ± 14.5	0.089
Total Operative Time (min), mean ± SD	345 ± 85	330 ± 80	310 ± 75	0.001
Estimated Blood Loss (mL), median (IQR)	400 (250–600)	350 (200–550)	300 (200–450)	<0.001
Pathological response (Post-neoadjuvant Groups Only)				
Pathological T Stage (AJCC 8th), n (%)				<0.001
ypT0	70 (29.8)	12 (4.0)	–	
ypT1–T2	110 (46.8)	90 (30.0)	–	
ypT3–T4	55 (23.4)	198 (66.0)	–	
Pathological N Stage (AJCC 8th), n (%)				<0.001
ypN0	170 (72.3)	120 (40.0)	–	
ypN+	65 (27.7)	180 (60.0)	–	
Mandard TRG, n (%)				<0.001
TRG 1	70 (29.8)	12 (4.0)	–	
TRG 2	65 (27.7)	33 (11.0)	–	
TRG 3-5 (partial)	100 (42.6)	255 (85.0)	–	
Pathological Response Categories, n (%)				<0.001
pCR	70 (29.8)	12 (4.0)	–	
mPR	135 (57.4)	45 (15.0)	–	
Partial Response (>10–50% viable)	60 (25.5)	114 (38.0)	–	
Poor/Minimal Response (>50% viable)	40 (17.0)	141 (47.0)	–	
Percentage of Viable Tumor Cells, median (IQR)	5 (0–20)	50 (30–70)	–	<0.001

NICT, neoadjuvant immunochemotherapy; NCT, neoadjuvant chemotherapy; US, upfront surgery; SD, standard deviation; IQR, interquartile range; AJCC, American Joint Committee on Cancer; TRG, tumor regression grade; pCR, pathologic complete response; mPR, major pathologic response.

### Major wound complications

**Figure 1:**
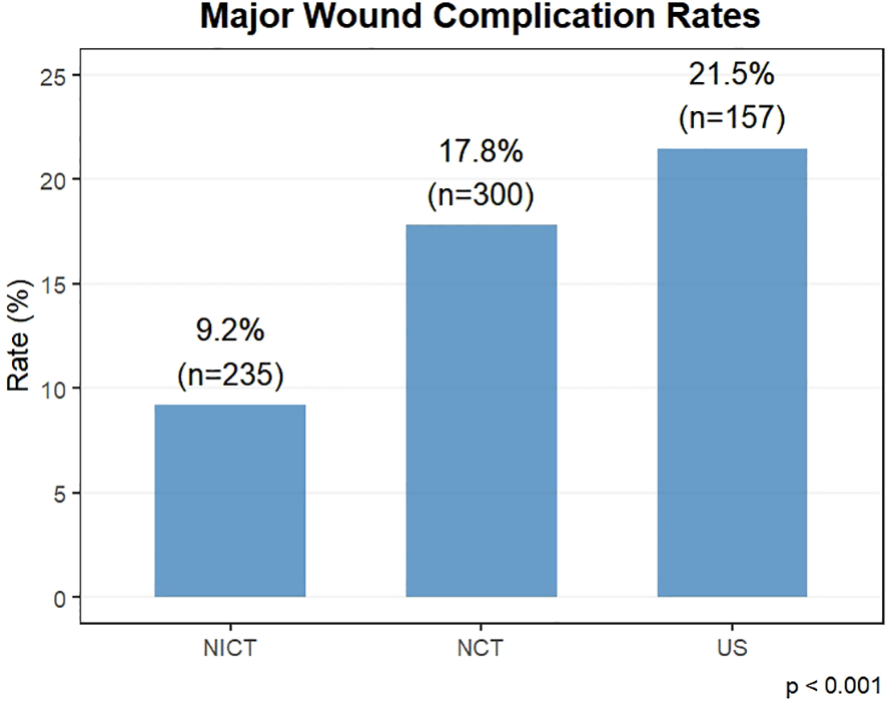
Comparison of major wound complication incidence across treatment cohorts.

The incidence of major wound healing complications was significantly different across the three cohorts (p<0.001). The lowest complication rate was observed in the NICT cohort (9.2%), followed by the NCT cohort (17.8%), and highest in the US cohort (21.5%) (**Figure 1**).

In the univariate analysis, treatment cohort, BMI <18.5 kg/m², smoking >30 pack-years, Charlson Comorbidity Index ≥2, advanced clinical T, N, and overall stage (IV vs. III), free flap reconstruction, longer operative time (>350 min), greater estimated blood loss (>500 mL), and poor/minimal pathological response were all significantly associated with an increased risk of major complications. After adjusting for potential confounders in the multivariable model, the NICT cohort maintained a significantly lower odds of major complications compared to both the US cohort [adjusted odds ratio (aOR) 0.40, 95% CI 0.22–0.71, p=0.001] and the NCT cohort (aOR 0.48, 95% CI 0.28–0.82, p=0.007). Independent risk factors for major wound complications identified in the final model included low BMI (<18.5 kg/m², aOR 1.65, p=0.027), heavy smoking (>30 pack-years, aOR 1.89, p=0.001), free flap reconstruction (aOR 1.65, p=0.014), high intraoperative blood loss (>500 mL, aOR 2.05, p<0.001), and poor/minimal pathological response to neoadjuvant therapy (aOR 2.10, p=0.002). Factors such as age, sex, comorbidity burden, and advanced clinical stage were not independent predictors after adjustment (**Table 2**).

**Table 2:** Univariate and multivariable logistic regression analysis of major wound complications (Clavien-Dindo ≥ III).

Variable	Category	Univariate		Multivariable	
		OR (95%CI)	p	aOR (95%CI)	p
Cohort	NICT vs. US	0.35 (0.20–0.62)	<0.001	0.40 (0.22–0.71)	0.001
	NCT vs. US	0.77 (0.48–1.24)	0.283	0.84 (0.51–1.39)	0.501
	NICT vs. NCT	0.45 (0.27–0.77)	0.003	0.48 (0.28–0.82)	0.007
Age (years)	≥65 vs. <65	1.32 (0.90–1.94)	0.158	1.20 (0.78–1.86)	0.404
Sex	Female vs. Male	1.18 (0.75–1.87)	0.472	1.12 (0.68–1.86)	0.649
BMI (kg/m²)	<18.5 vs. ≥18.5	1.81 (1.20–2.72)	0.005	1.65 (1.06–2.58)	0.027
Smoking (pack-years)	>30 vs. ≤30	2.05 (1.43–2.95)	<0.001	1.89 (1.28–2.80)	0.001
Alcohol Use	Yes vs. No	1.10 (0.76–1.59)	0.603	1.05 (0.70–1.58)	0.813
CCI	≥2 vs. 0–1	1.62 (1.12–2.36)	0.011	1.31 (0.87–1.99)	0.197
Primary Tumor Site	Oral Tongue vs. Others	0.88 (0.60–1.30)	0.530	0.92 (0.61–1.40)	0.710
Clinical T Stage	T3–T4 vs. T1–T2	1.95 (1.28–2.96)	0.002	1.52 (0.96–2.40)	0.076
Clinical N Stage	N2–N3 vs. N0–N1	1.70 (1.17–2.47)	0.006	1.32 (0.87–2.00)	0.195
Clinical Stage	IV vs. III	1.78 (1.22–2.60)	0.003	1.44 (0.95–2.19)	0.085
Differentiation	Poor vs. Well/Moderate	1.45 (0.98–2.16)	0.066	1.30 (0.85–2.00)	0.231
Neck Dissection Type	MRND vs. Selective	1.35 (0.94–1.94)	0.102	1.22 (0.82–1.81)	0.331
Reconstructive	Free Flap vs. Others	1.90 (1.32–2.73)	<0.001	1.65 (1.11–2.45)	0.014
Flap Ischemia Time	>85 vs. ≤85	1.40 (0.96–2.03)	0.080	1.25 (0.83–1.87)	0.285
Total Operative Time	>350 vs. ≤350	1.68 (1.17–2.42)	0.006	1.40 (0.94–2.09)	0.100
Estimated Blood Loss	>500 vs. ≤500	2.35 (1.63–3.39)	<0.001	2.05 (1.38–3.04)	<0.001
Pathological Response	Poor/Minimal vs. mPR	2.50 (1.62–3.85)	<0.001	2.10 (1.31–3.37)	0.002

NICT, neoadjuvant immunochemotherapy; NCT, neoadjuvant chemotherapy; US, upfront surgery; mPR, major pathologic response; HR, Hazard Ratio; aHR, Adjusted Hazard Ratio; CI, Confidence Interval; CCI, Charlson Comorbidity Index; MRND, modified radical neck dissection.

For pairwise cohort comparisons, the reference groups are as follows, “NICT vs. US” uses US as reference; “NCT vs. US” uses US as reference; “NICT vs. NCT” uses NCT as reference. All comparisons were derived from the same multivariable logistic regression model with categorical cohort variable entered as three-level factor and parameterized using appropriate contrast coding.

### Time to complete wound sealing

**Figure 2:**
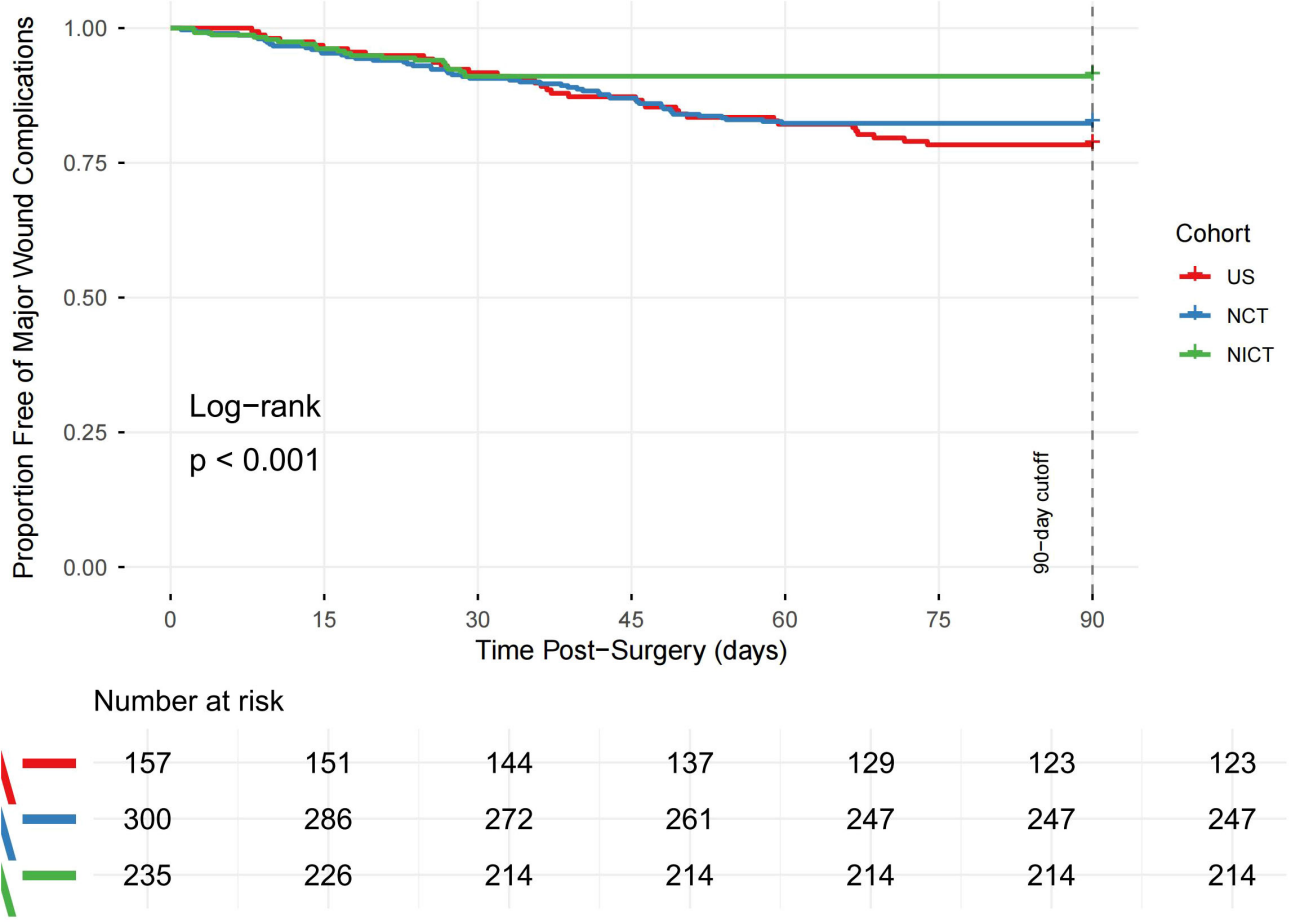
Kaplan-Meier analysis of time to complete wound sealing by treatment cohort.

The Kaplan-Meier curve (**Figure 2**) demonstrated a significantly faster wound healing trajectory for the NICT cohort compared to both the NCT and US cohorts (log-rank p < 0.001). In the univariate Cox analysis, factors associated with a significantly shorter time to wound sealing (indicated by a hazard ratio >1) included belonging to the NICT cohort, higher BMI (≥18.5 kg/m²), lighter smoking history (≤30 pack-years), less advanced clinical stage (III vs. IV), reconstruction with methods other than free flap, lower estimated blood loss (≤500 mL), and achieving a mPR. After multivariable adjustment, receiving NICT remained the strongest independent predictor of accelerated wound sealing. Patients in the NICT cohort achieved complete wound sealing 1.85 times faster than those in the US cohort [adjusted hazard ratio (aHR) 1.85, 95% CI 1.42–2.41, p<0.001] and 1.54 times faster than those in the NCT cohort (aHR 1.54, 95% CI 1.20–1.98, p=0.001). Other independent factors associated with faster healing in the final model included higher BMI (aHR 1.25, p=0.049), lighter smoking (aHR 1.35, p=0.006), less advanced clinical stage (aHR 1.25 for stage III vs. IV, p=0.049), use of reconstruction methods other than free flap (aHR 1.38, p=0.004), lower estimated blood loss (aHR 1.45, p=0.001), and achieving a major pathological response (aHR 1.58, p=0.001) (**Table 3**).

**Table 3:** Univariate and multivariable cox proportional hazards analysis of time to complete wound sealing.

Variable	Category	Univariate		Multivariable	
		HR (95%CI)	p	aHR (95%CI)	p
Cohort					
	NICT vs. US	1.80 (1.38–2.35)	<0.001	1.85 (1.42–2.41)	<0.001
	NCT vs. US	1.18 (0.91–1.53)	0.213	1.20 (0.92–1.56)	0.176
	NICT vs. NCT	1.52 (1.19–1.95)	0.001	1.54 (1.20–1.98)	0.001
Age (years)	≥65 vs. <65	0.82 (0.66–1.02)	0.075	0.87 (0.69–1.09)	0.223
Sex	Female vs. Male	0.95 (0.73–1.24)	0.712	0.98 (0.75–1.28)	0.871
BMI (kg/m²)	≥18.5 vs. <18.5	1.30 (1.04–1.62)	0.022	1.25 (1.00–1.57)	0.049
Smoking (pack-years)	≤30 vs. >30	1.40 (1.13–1.73)	0.002	1.35 (1.09–1.67)	0.006
Alcohol Use	No vs. Yes	1.10 (0.88–1.37)	0.395	1.08 (0.86–1.35)	0.513
CCI	0–1 vs. ≥2	1.22 (0.98–1.52)	0.071	1.15 (0.92–1.44)	0.224
Primary Site	Tongue vs. Others	1.05 (0.85–1.30)	0.637	1.02 (0.82–1.27)	0.851
Clinical T Stage	T1/2 vs. T3/4	1.25 (1.00–1.56)	0.053	1.18 (0.94–1.48)	0.158
Clinical N Stage	N0/1 vs. N2/3	1.28 (1.03–1.59)	0.025	1.22 (0.98–1.52)	0.079
Clinical Stage	III vs. IV	1.33 (1.07–1.65)	0.011	1.25 (1.00–1.56)	0.049
Differentiation	Others vs. Poor	1.15 (0.92–1.44)	0.232	1.10 (0.87–1.38)	0.430
Neck Dissection	Selective vs. MRND	1.18 (0.96–1.45)	0.112	1.14 (0.92–1.41)	0.242
Reconstruction	Others vs. Free Flap	1.45 (1.17–1.79)	<0.001	1.38 (1.11–1.72)	0.004
Flap Ischemia Time	≤85 vs. >85	1.20 (0.97–1.48)	0.091	1.16 (0.94–1.44)	0.172
Total Operative Time	≤350 vs. >350	1.30 (1.05–1.61)	0.016	1.22 (0.98–1.52)	0.076
Estimated Blood Loss	≤500 vs. >500	1.52 (1.23–1.88)	<0.001	1.45 (1.17–1.80)	0.001
Pathological Response	mPR vs. Poor/Minimal	1.65 (1.28–2.13)	<0.001	1.58 (1.22–2.05)	0.001

NICT, neoadjuvant immunochemotherapy; NCT, neoadjuvant chemotherapy; US, upfront surgery; mPR, major pathologic response; HR, Hazard Ratio; aHR, Adjusted Hazard Ratio; CI, Confidence Interval; CCI, Charlson Comorbidity Index; MRND, modified radical neck dissection.

For pairwise cohort comparisons, the reference groups are as follows, “NICT vs. US” uses US as reference; “NCT vs. US” uses US as reference; “NICT vs. NCT” uses NCT as reference. All comparisons were derived from the same multivariable logistic regression model with categorical cohort variable entered as three-level factor and parameterized using appropriate contrast coding.

### Postoperative clinical outcomes

Patients in the NICT cohort demonstrated superior recovery metrics compared to both control groups. The median postoperative length of stay was significantly shorter for the NICT cohort (10 days, IQR 8–14) compared to the NCT cohort (14 days, IQR 10–18) and the US cohort (15 days, IQR 11–19) (p < 0.001). Similarly, healthcare utilization measures were significantly lower for the NICT cohort, with a 30-day readmission rate of 6.4% (vs. 10.0% in NCT and 12.7% in US, p=0.048) and an unplanned reoperation rate of 4.3% (vs. 8.3% in NCT and 11.5% in US, p=0.012). Furthermore, the time to initiation of adjuvant radiotherapy was also significantly shorter in the NICT cohort (median 42 days) than in the NCT cohort (50 days, p=0.001), although the difference compared to the US cohort (45 days) was less pronounced (**Table 4**).

**Table 4:** Postoperative outcomes by cohort.

Outcome Measure	NICT Cohort (n=235)	NCT Cohort (n=300)	US Cohort (n=157)	p-value
Postoperative LOS (days),	10 (8–14)	14 (10–18)	15 (11–19)	<0.001
30-day Readmission Rate	15 (6.4%)	30 (10.0%)	20 (12.7%)	0.048
Unplanned Reoperation	10 (4.3%)	25 (8.3%)	18 (11.5%)	0.012
Time to Adjuvant RT (days),	42 (35–50)	50 (42–60)	45 (38–55)	0.001

NICT, neoadjuvant immunochemotherapy; NCT, neoadjuvant chemotherapy; US, upfront surgery; LOS, length of hospital stay; RT, radiotherapy.

Data are presented as median (interquartile range) for continuous variables (Postoperative LOS and Time to Adjuvant RT) and as n (%) for categorical variables (30-day Readmission Rate and Unplanned Reoperation).

Postoperative LOS and Time to Adjuvant RT were compared using the Kruskal–Wallis test; 30-day Readmission Rate and Unplanned Reoperation were compared using Pearson’s Chi-square test.

LOS, Length of Stay; RT, Radiotherapy.

### Risk factors for complications within the NICT cohort

To delineate factors specifically influencing wound healing outcomes in the context of NICT, a dedicated risk factor analysis was performed within the NICT cohort (**Table 5**). In multivariable logistic regression, five independent risk factors for major wound complications were identified. The strongest predictor was a poor/minimal pathological response (aOR 3.40, 95% CI 1.78–6.50, p<0.001), reaffirming the critical link between tumor regression and postoperative healing. Other significant modifiable risk factors included a shorter treatment-to-surgery interval (<28 days, aOR 2.30, p=0.010), heavy smoking (>30 pack-years, aOR 2.45, p=0.007), high intraoperative blood loss (>500 mL, aOR 2.60, p=0.004), and the use of free flap reconstruction (aOR 2.15, p=0.021). Interestingly, age ≥65 years also emerged as an independent risk factor within this cohort (aOR 2.10, p=0.024).

**Table 5:** Risk factor analysis for major wound complications within the NICT cohort.

Variable	Category	Univariate		Multivariable	
		OR	p	aOR (95%CI)	p
Age (years)	≥65 vs. <65	1.95 (0.85–4.48)	0.116	2.10 (1.10–4.01)	0.024
Sex	Female vs. Male	1.20 (0.48–3.01)	0.704	1.15 (0.60–2.20)	0.674
BMI (kg/m²)	<18.5 vs. ≥18.5	1.80 (0.75–4.32)	0.187	1.70 (0.82–3.52)	0.153
Smoking (pack-years)	>30 vs. ≤30	2.55 (1.15–5.67)	0.021	2.45 (1.28–4.68)	0.007
Treatment–Surgery Interval	<28 vs. ≥28 days	2.45 (1.18–5.09)	0.017	2.30 (1.22–4.34)	0.010
Number of NICT Cycles	>3 vs. ≤3 cycles	1.30 (0.60–2.82)	0.505	1.25 (0.68–2.30)	0.475
Pathological Response	Poor/Minimal vs. mPR	3.65 (1.72–7.74)	<0.001	3.40 (1.78–6.50)	<0.001
Free Flap Reconstruction	Yes vs. No	2.20 (1.02–4.75)	0.045	2.15 (1.12–4.12)	0.021
Estimated Blood Loss (mL)	>500 vs. ≤500	2.70 (1.25–5.83)	0.011	2.60 (1.35–5.01)	0.004
Clinical Stage	IV vs. III	1.60 (0.73–3.52)	0.240	1.45 (0.78–2.70)	0.238
Charlson Index	≥2 vs. 0–1	1.50 (0.68–3.31)	0.314	1.35 (0.71–2.56)	0.363

NICT, neoadjuvant immunochemotherapy; mPR, major pathologic response; OR, odds ratio; aOR, adjusted odds ratio; CI, Confidence Interval.

### Subgroup and sensitivity analyses

A treatment-to-surgery interval of 28–35 days was associated with significantly lower odds of major complications compared to shorter intervals (aOR 0.48, 95% CI 0.27–0.85, p=0.012), particularly in patients receiving NICT. Poor/minimal pathological response remained an independent predictor of complications after adjustment (aOR 2.95, 95% CI 1.70–5.12, p<0.001). Stratification by reconstructive method showed that the wound-healing advantage of NICT was consistent whether free flap or other reconstruction was used (interaction p>0.4), indicating the benefit persists across surgical complexities. In sensitivity analysis, NICT was also strongly associated with a higher likelihood of complete healing by postoperative day 14 (aOR 2.40, 95% CI 1.62–3.56, p<0.001) (**Supplementary Table 1**).

## Discussion

This large, single-center retrospective cohort study demonstrates that patients with locally advanced OSCC who receive NICT experience significantly fewer major wound complications and achieve faster complete wound sealing compared to those receiving NCT alone or undergoing US. These findings support our primary hypothesis that the immunomodulatory and potent antitumor effects of NICT create a more favorable milieu for postoperative healing. The 9.2% complication rate in the NICT cohort, which is less than half that of the US cohort, along with a 1.85-fold faster time to wound closure, suggests that NICT not only improves oncological downstaging but also confers a tangible benefit to surgical recovery, translating into shorter hospital stays and lower rates of unplanned reoperations.

Our observation of a markedly reduced complication rate (9.2%) in the NICT group—less than half that of the US cohort—is particularly compelling given the complexity of OSCC reconstruction. This aligns with evidence suggesting immune checkpoint inhibitors (ICIs) may modulate inflammatory pathways to promote tissue repair. A recent editorial (11) explicitly questions the safety of neoadjuvant immunotherapy before HNSCC surgery. Our data provide clinical corroboration that its expansion may be oncologically rational and surgically safe, potentially advantageous. This conclusion is supported by a multi-cancer NCDB analysis finding no association between neoadjuvant immunotherapy and increased major surgical morbidity (12). That study acknowledges limitations but lends robustness to the negative finding, emphasizing that understanding surgical safety is crucial as immunotherapy use grows—a sentiment echoed by our OSCC-specific findings.

Similarly, a focused retrospective matched-cohort study of OSCC patients receiving neoadjuvant pembrolizumab reported no significant increase in 30-day postoperative adverse events versus matched historical controls (13). Using propensity-score matching and McNemar testing to minimize bias, it found the overall reconstructive burden remained high without increased complications, supporting the view that NICT does not inherently compromise complex reconstruction. One patient even avoided free-flap reconstruction due to tumor regression, a phenomenon also seen in our cohort. However, contradictory evidence exists. A separate single-institution study reported a statistically significant increase in recipient-site wound complications after ICI exposure, particularly for skin/scalp (p=0.005), with a 31% complication rate including dehiscence, fistula, and infection (14). While methodological differences may explain the discrepancy, this highlights that ICI impact on healing is likely context-dependent, influenced by anatomical site, reconstructive technique, dosing, and patient factors like prior radiation.

Indeed, the potential for severe complications cannot be dismissed. A case series describing four patients on anti–PD-1 monoclonal antibodies who developed osteoradionecrosis and complex wound complications highlights the real, albeit rare, risks (15). All four required extensive surgical intervention at tertiary centers, though notably, they ultimately recovered and resumed immunotherapy. This suggests that while catastrophic complications can occur particularly in previously irradiated fields, they are manageable with prompt recognition and aggressive surgical care. Critically, none of these cases involved neoadjuvant use in treatment-naïve patients; rather, they occurred in recurrent or metastatic settings, often after prior chemoradiation. This distinction is vital: the wound-healing milieu in a previously irradiated, fibrotic bed differs fundamentally from that in a treatment-naïve surgical field. Our cohort consisted exclusively of treatment-naïve, locally advanced OSCC patients, which may partly explain our favorable outcomes.

Further support for the safety of neoadjuvant ICIs comes from early-phase clinical trials, which demonstrated safety and tolerability, without reported surgical delays, complications, or osteoradionecrosis in the neoadjuvant setting (16). These prospective data, though limited in scale, align with our retrospective findings and with the NCDB-based multi-cancer analysis (12), collectively building a case for the feasibility of integrating ICIs into preoperative regimens.

A recent meta-analysis of six randomized trials involving 2,941 patients with resectable non-small cell lung cancer examined perioperative ICIs and found that while preoperative ICI therapy may increase wound complications, postoperative administration did not (17). This temporal nuance is critical: it implies that the timing of ICI exposure relative to surgery is a key determinant of wound outcomes. In our study, NICT was administered in a defined neoadjuvant window (typically 2-3 cycles) with a planned interval before surgery, allowing for immune activation without coinciding with the peak inflammatory phase of wound healing. This strategic scheduling may mitigate potential interference with early healing processes, such as hemostasis and granulation tissue formation, which are highly sensitive to dysregulated inflammation.

Moreover, the biological plausibility of improved healing with NICT lies in its dual action: potent antitumor efficacy and immunomodulation. By reducing tumor burden preoperatively, NICT may decrease local tissue invasion, inflammation, and lymphatic obstruction—all of which impair wound perfusion and increase infection risk (18). Simultaneously, ICIs may recalibrate the postoperative immune response away from a pro-fibrotic, chronic inflammatory state toward a more regulated, pro-reparative phenotype. As noted in our manuscript, emerging data suggest that neoadjuvant immunotherapy could also favorably modulate the postoperative wound microenvironment, possibly leading to enhanced healing. This hypothesis is consistent with preclinical models showing that PD-1/PD-L1 blockade can enhance macrophage polarization toward an M2 reparative phenotype and improve collagen deposition (10).

In synthesizing these findings, a nuanced picture emerges: NICT is not uniformly detrimental to wound healing, as its effects appear to be modulated by multiple factors, including timing—where preoperative administration requires careful scheduling to avoid overlap with critical healing phases—tumor biology and response, wherein significant regression may reduce surgical complexity (19) and improve tissue quality, anatomic site, with mucosal sites like the oral cavity responding differently than cutaneous or irradiated fields, combination therapy, as chemotherapy added to ICI may have synergistic or mitigating effects on inflammation, and patient history, in which prior radiation or recurrent disease dramatically increases complication risk (20). Our study contributes uniquely by demonstrating not just non-inferiority, but superiority of NICT in terms of wound outcomes—a finding not previously reported in the literature. While others have shown no increase in complications, we show a significant reduction and accelerated healing, which may be attributed to the specific regimen, the homogeneous OSCC population, or the meticulous surgical and perioperative care at our institution. Interestingly, a treatment-to-surgery interval of <28 days significantly increases complication risk, aligning with subgroup findings that 28–35 days offers the lowest risk. This suggests an optimal window exists that balances immune activation with the subsidence of inflammation. Furthermore, the strong association between poor pathological response and complications reinforces the link between tumor regression and improved tissue recovery. Age ≥65 emerged as a unique independent risk factor, indicating that older patients require heightened physiological optimization (21). The consistent wound-healing advantage of NICT across different reconstruction methods confirms that its benefit is not negated by surgical complexity. A low BMI (<18.5 kg/m²) emerged as a robust independent predictor of major wound complications and was significantly associated with a slower time to complete wound sealing. This association likely reflects the physiological impact of malnutrition and cancer cachexia, which are prevalent in locally advanced OSCC due to tumor-induced dysphagia and odynophagia (22). Biologically, wound healing is an anabolic process; a depleted nutritional state limits the bioavailability of essential substrates required for the proliferative phase, particularly for fibroblast function and collagen synthesis, thereby compromising the tensile strength of the surgical closure (23). Furthermore, nutritional deficiency is intrinsically linked to immune dysregulation, potentially impairing local neutrophil chemotaxis and increasing susceptibility to surgical site infections. From a technical perspective, patients with significant cachexia often present with reduced subcutaneous adipose tissue, resulting in thinner cutaneous and mucosal flaps that may be more vulnerable to ischemic insult during complex reconstruction (24).

Nonetheless, limitations must be acknowledged. As a single-center retrospective study, our findings require validation in prospective, multicenter trials. The sample size, while substantial for a single institution, may lack power to detect rare but serious complications. Additionally, long-term functional outcomes, such as speech, swallowing, and cosmesis, were not assessed, though complete wound sealing is a necessary prerequisite for optimal function.

In conclusion, NICT in locally advanced OSCC is not only oncologically effective but also surgically beneficial, significantly reducing major wound complications and accelerating complete wound healing compared to neoadjuvant chemotherapy or upfront surgery. These superior outcomes, driven by the regimen’s dual antitumor and immunomodulatory effects, are further optimized by key modifiable factors such as an adequate treatment-to-surgery interval (28–35 days) and achieving a major pathological response. Importantly, the benefit of NICT persists irrespective of reconstruction type, reinforcing its practical applicability across varying surgical complexities. These findings challenge previous concerns regarding perioperative safety and support the integration of NICT—guided by tailored patient selection and optimized timing—into perioperative care pathways to enhance both oncological and surgical recovery for patients with OSCC.

## Data Availability

The original contributions presented in the study are included in the article/**Supplementary Material**. Further inquiries can be directed to the corresponding author.
