# Supplementary material for: Immunomodulation in healing: neoadjuvant immunochemotherapy reduces major wound complications and accelerates recovery in oral cancer surgery
Source: Front Immunol. 2026 Feb 27;17:1768004. doi: 10.3389/fimmu.2026.1768004 (PMC12982033; doi:10.3389/fimmu.2026.1768004)
Supplement: Supplementary file 1 [file DataSheet1.doc]

### ****Supplementary Table 1: Results of Subgroup and Sensitivity Analyses for Major Wound Complications****

| ****Analysis Type**** | ****Subgroup / Comparison**** | ****Effect Estimate (95% CI)**** | ****p**** | ****Interaction p-value**** |
| --- | --- | --- | --- | --- |
| ****Treatment-to-Surgery Interval**** | 28–35 days vs. ≤21 days | aOR 0.48 (0.27–0.85) | 0.012 | – |
|  | 28–35 days vs. 22–27 days | – | – | – |
| ****Pathological Response**** | Partial Response vs. mPR | aOR 1.95 (1.12–3.40) | 0.018 | – |
|  | Poor/Minimal Response vs. mPR | aOR 2.95 (1.70–5.12) | <0.001 | – |
| ****Type of Reconstruction**** | ****Free Flap**** |  |  |  |
|  | NICT vs. US | aOR 0.32 (0.16–0.64) | 0.001 | 0.412 |
|  | NICT vs. NCT | aOR 0.42 (0.22–0.80) | 0.008 | 0.528 |
|  | ****Other Reconstruction**** |  |  |  |
|  | NICT vs. US | aOR 0.45 (0.21–0.95) | 0.036 |  |
|  | NICT vs. NCT | aOR 0.52 (0.27–1.02) | 0.058 |  |
| ****Early Healing (Day 14)**** | NICT vs. US | aOR 2.40 (1.62–3.56) | <0.001 | – |
|  | NICT vs. NCT | aOR 1.85 (1.25–2.74) | 0.002 | – |
|  | NCT vs. US | aOR 1.30 (0.90–1.88) | 0.172 | – |

****Abbreviations:**** aOR = adjusted odds ratio; CI = confidence interval; mPR = major pathological response; NICT = neoadjuvant immunotherapy with chemotherapy; NCT = neoadjuvant chemotherapy; US = upfront surgery.

**Adjustment variables**: All adjusted odds ratios (aORs) presented were derived from multivariable logistic regression models that controlled for baseline and surgical covariates, including age, sex, body mass index, smoking pack-years, Charlson Comorbidity Index, clinical stage, type of reconstruction, operative time, and estimated blood loss (as detailed in the Methods section).

**Interpretation of interaction p-values**: The interaction p-values test whether the treatment effect (NICT vs. US or NICT vs. NCT) differs significantly between the reconstruction subgroups (Free Flap vs. Other Reconstruction). A non-significant interaction (e.g., p = 0.412 or 0.528) suggests that the benefit of NICT is consistent across both types of reconstruction.

**Dashes (—)** indicate that an interaction test or subgroup comparison was not applicable or not performed for that specific analysis.
